# Adjuvant endocrine therapy in estrogen-low breast cancer in the era of CDKI: are we at another uncertain crossroads?

**DOI:** 10.3389/fonc.2026.1636348

**Published:** 2026-05-07

**Authors:** Maria Rosaria Valerio, Giuseppa Scandurra, Martina Greco, Chiara Mesi, Vittorio Gebbia, Daniela Sambataro

**Affiliations:** 1 Medical Oncology Unit, Policlinico Universitario, University of Palermo, Palermo, Italy; 2 Medical Oncology Unit, Ospedale Cannizzaro, Catania, Italy; 3 Department of Medicine and Surgery , Kore University of Enna, Enna, Italy; 4 Medical Oncology Unit, Casa di Cure (CdC) Torina, Palermo, Italy; 5 Medical Oncology Unit, Ospedale Umberto I, Enna, Italy

**Keywords:** adjuvant therapy, biomarkers, CCND1, CDK4/6 inhibitors, endocrine therapy, estrogen receptor-low breast cancer, MONARCH E, NATALEE

## Abstract

Estrogen receptor-low breast cancer is defined as ER expression of 1–10% by immunohistochemistry. It occupies a debated space between luminal and triple-negative disease. The CDK4/6 inhibitors abemaciclib and ribociclib have set new standards for high-risk HR+/HER2-/early breast cancer. However, neither trial stratified patients by ER expression levels. This leaves the ER-low subgroup without dedicated prospective evidence. This review synthesizes data from adjuvant CDK4/6 inhibitor trials, clinical and molecular studies of ER-low BC, biomarker evidence, regulatory context, and chemotherapy/immunotherapy data, with specific attention to the ER-low subgroup and a structured framework for clinical decision-making. ER-low breast cancer is mostly non-luminal (about 75–80% basal-like). Its molecular features, neoadjuvant chemotherapy response rates, and survival outcomes are closer to ER-negative/triple negative breast cancer than ER-high luminal cancer. Both MONARCH E and NATALEE trials enrolled ER-low patients, using the ≥1% positivity threshold. Exploratory subgroup data suggest a numerically consistent benefit from CDK4/6 inhibitors. However, confidence intervals are wide, and formal statistical significance is not shown. PAL trials showed no benefit. This is linked to its specific pharmacology rather than a class effect. CDK4/6 inhibitor activity in ER-low disease appears to result from ER-independent RB pathway mechanisms, CCND1 amplification, and immunomodulatory effects. Frequent RB1 loss, reduced endocrine sensitivity, and cell-cycle control by CDK2/cyclin E counterbalance this activity. ER-low patients who meet high-risk trial eligibility criteria may receive adjuvant CDK4/6 inhibitors when luminal biomarkers support HR+ biology but must be counseled about the evidence gap. Molecular subtyping (PAM50), RB1 assessment, PR status, germline BRCA testing, and multidisciplinary tumor board review are mandatory. Dedicated prospective trials with ER-low as a pre-specified stratum are urgently needed.

## 1 Introduction

Breast cancer (BC) is the most prevalent malignancy among women globally and remains a leading cause of cancer-related mortality. Classification of BC has evolved through histological and molecular subtyping, particularly with the adoption of the 50-gene PAM50 signature, which delineates luminal A, luminal B, HER2-enriched, basal-like, and normal-like subtypes (1, 2). Approximately 70% of cases are hormone receptor-positive (HR+) and human epidermal receptor-2-negative (HER2-). Advances in endocrine therapy (ET) have shaped treatment strategies by specifically targeting hormone-driven tumor growth (3).

Cyclin-dependent kinase 4 and 6 inhibitors (CDK4/6i), including palbociclib (PAL), ribociclib (RIB), and abemaciclib (ABE), have significantly altered the treatment paradigm for metastatic HR+/BC by inhibiting the G1/S cell cycle transition and enhancing the cytostatic effects of ET (4). Multiple phase III trials have demonstrated consistent improvements in progression-free survival (PFS) and, for RIB and ABE, overall survival (OS), supporting further evaluation in the adjuvant setting (5, 6). The MONARCH E and NATALEE phase III trials have established CDK4/6i in combination with ET as the standard adjuvant therapy for patients with high-risk, early-stage HR+/HER2- BC (4–6).

Estrogen receptor-low (ER-low) BC is characterized by low ER protein expression, typically defined as 1–9% or 10% positivity on immunohistochemistry (IHC). Historically, this threshold has classified tumors as ER+ and rendered them eligible for ET. However, ER-low tumors may exhibit biological behavior more like ER-negative or triple-negative breast cancer (TNBC) (7, 8). The 2020 ASCO/CAP guideline introduced the term ‘ER low positive’ to describe this subgroup, emphasizing the uncertain benefit of ET and the need for individualized assessment (7). The prevalence of ER-low BC ranges from 1.6% to 7% in large cohorts (3, 8, 9).

The benefit of adjuvant CDK4/6i in ER-low tumors remains an urgent and unresolved clinical question. Patients with ER-low disease may be undertreated if luminal-directed therapies are presumed to be as effective as in ER-high cases or overtreated with targeted agents if cell cycle dependency is lacking. The phase III MONARCH E and NATALEE trials enrolled patients based on institutional HR+ status, thereby including ER-low individuals; however, neither trial reported ER-level subgroup analyzes sufficient to definitively address this issue (5, 6).

This review examines the biological rationale for CDK4/6 inhibition in ER-low disease, evaluates clinical evidence from major adjuvant CDK4/6i trials with a focus on ER-low subgroups, and assesses biomarker data relevant to patient selection. Additionally, it considers the roles of chemotherapy and immunotherapy. The review concludes by proposing a structured decision-making framework for ER-low patients eligible for clinical trials.

## 2 Biological background

### 2.1 The CDK4/6–cyclin D–RB axis

The cell cycle is regulated by a sequence of cyclin-dependent kinase activations. When CDK4 and CDK6 bind to D-type cyclins, especially CCND1, they phosphorylate RB1 (10, 11). This phosphorylation releases E2F transcription factors, helping the cell enter S-phase. In classical luminal tumors, estrogen signaling via ERα increases CCND1 transcription, thereby sustaining CDK4/6 activation. CDK4/6 inhibitors (CDK4/6i) block the ATP-binding sites of CDK4 and CDK6 (10/11). As a result, RB1 phosphorylation is prevented, and G1 cell cycle arrest is induced.

CDK4/6 inhibition induces cell cycle arrest and immunomodulatory effects (10, 11). These include upregulation of antigen presentation, interferon signaling, and PD-L1 expression, which may enhance antitumor immunity (12). ABE also inhibits CDK9 and CLK2 (13). Its continuous dosing schedule may yield greater anti-proliferative effects than the intermittent schedules used PAL and RIB (13). Additionally, ABE penetrates the central nervous system, which may be relevant for micrometastatic disease. RB1 function must be intact for CDK4/6i to be effective (13). Tumors lacking RB1 cannot undergo G1 arrest, even with CDK inhibition.

### 2.2 Molecular landscape of ER-low breast cancer

ER-low BC has significant molecular heterogeneity. Gene expression profiling shows ER-low tumors include many intrinsic subtypes. Most cluster with basal-like or luminal B. A minority are similar to classical luminal A subtypes (14, 15). Iwamoto et al. found ESR1 mRNA positivity in only 24% of ER 1–9% tumors, compared to 92% of ER >10% tumors (14). Among ER-low cases, 48% were basal-like and only 8% were luminal B (14). Higgins et al. used the Cancer Genome Atlas (TCGA) and the National Cancer Database. They discovered 82.6% of ER-low+/HER2− tumors were basal-like. Additionally, 67% had an Oncotype DX Recurrence Score of 26 or higher (15). Another study found 45% of ER-low tumors were ER-negative when re-examined with quantitative PCR, especially in core biopsy samples (16).

ER-low disease shows distinct genomic characteristics (17, 18). These include increased TP53 mutation frequency, reduced PIK3CA mutations, and greater genomic instability. CCND1 amplification directly drives CDK4/6 activation independent of ER signaling. It is present in a subset of ER-low tumors and may preserve sensitivity to CDK4/6i. In contrast, RB1 loss is seen more often in ER-low tumors (20–30%) than in ER-high luminal cancers (less than 5%). This suggests a significant subset of patients may have CDK4/6i resistance. Spatial transcriptomics identified distinct compartments in ER+/BC. These are estrogen-responsive, marked by rapid cell division and high Ki67 protein levels; hypoxia-induced, shaped by low oxygen conditions; and inflammation-related, associated with immune system activity (18). The Ki67-high compartment promotes estrogen-independent growth and activates the CDK4/6 pathway, controlling cell division. This compartment, not ER expression alone, seems to determine the response to cell-cycle therapies targeting replication.

### 2.3 Immune landscape and endocrine sensitivity

Massa et al. studied the tumor immune microenvironment in 921 HER2-/ER-low+ patients (ER 1–9%) (17). They found that median tumor-infiltrating lymphocyte (TIL) levels, the CD8/FOXP3 ratio, and PD-L1 positivity were similar in ER-low+ and ER-negative tumors, and both were much higher than in ER-intermediate tumors. The basal-like subtype and immune gene expression profiles were also similar in these groups (17). High TIL scores (≥30%) were associated with improved 5-year relapse-free survival in ER-low+/BC (78.6% vs 66.2%; p=0.033), suggesting that immune checkpoint blockade may benefit these patients. These results highlight the need to study how the immune landscape and endocrine therapies interact to better understand treatment response.

The effectiveness of CDK4/6i combined with ET in ER+/BC depends on endocrine therapy lowering ER-driven CCND1 expression (19). In ER-low tumors, this effect may be weaker, potentially limiting the combined benefit. Still, CDK4/6i can slow tumor growth on their own through ER-independent pathways, and their effects on the immune system may help even when ER levels are low. Preclinical studies in ER-low cell lines also show that ABE still reduces cell growth at low ER levels, though this effect is usually weaker than in ER-high models (2, 3).

## 3 CDK4/6 inhibitors: distinct profiles relevant to ER-low disease

**Table 1** synthesizes the principal studies evaluating ER-low BC outcomes across different therapeutic contexts, designs, and settings (7, 8, 12, 14, 20–29).

**Table 1:** Summary of key clinical studies on ER-low breast cancer outcomes and treatment implications.

Reference	Type of study	Setting	ER status	Results	Suggestions	Potential pitfalls	Conclusions
EBCTCG (20)	Meta-analysis	Early BC	ER <1/3 (H-score)	Tamoxifen benefit correlated with ER level; negligible in ER-poor tumors	ET benefit tracks ER expression — foundational evidence	Pre-2010 cohorts; heterogeneous assays	No benefit
Iwamoto et al. (14)	Retrospective	Early BC	IHC 1–10%	Only 24% ER 1–9% tumors were ER+ by mRNA; 48% basal-like	mRNA testing may better stratify endocrine sensitivity	Single institution	No benefit/TNBC-like
Fujii et al. (21)	Retrospective	Early BC	IHC 1–9%	ER 1–9% HER2− patients: little ET benefit; TNBC-like outcomes	Treat ER-low HER2− similarly to TNBC	Small cohort; single center	No benefit/TNBC-like
Deyarmin et al. (22)	Retrospective	Early BC	IHC 1–9% vs ≥10%	Most ER 1–9% tumors: basal-like; very few luminal	Molecular subtyping essential to complement IHC	Retrospective; TMA IHC	No benefit/TNBC-like
Villegas et al. (23)	Retrospective	Neoadjuvant EBC	HR low-positive (1–9%)	Higher pCR rates with NAC vs ER-high; comparable to TNBC	Chemo ± immunotherapy preferred over ET-based strategies	Pooled trial data; heterogeneous regimens	Partial/context-dependent
Poon et al. (24)	Retrospective	Early BC	ER 1–10%	ER-low: basal-like, worse prognosis; AJCC staging underestimates risk	AJCC downstaging may not apply; undertreatment risk	TMA IHC; limited follow-up	No benefit/TNBC-like
Acs et al. (25)	Retrospective	Early BC (TNBC treatment)	ER 1–9% (≥10% threshold era)	ER-low treated as TNBC: OS and recurrence comparable to ER-zero	Supports ≥10% threshold; TNBC management appropriate	Limits generalizability to 1% threshold settings	No benefit/TNBC-like
Voorwerk et al. (26)	Retrospective	Early BC (sTIL)	ER 1–10%	Intermediate-high sTILs, CD8+, PD-L1: between ER-zero and ER-high	Subset may respond to immune checkpoint blockade	Enriched cohort; small sample	Uncertain/heterogeneous
Benefield et al. (27)	Retrospective	Early BC	IHC 1–9%	Higher ER-low prevalence in Black women; worse survival independent of treatment	Equity implications for ER-low classification	Registry data; treatment heterogeneity	Uncertain/heterogeneous
Royal Marsden (28)	Retrospective	Advanced BC	ER Allred ≤5	CDK4/6i + ET: mPFS 13.9 months 1st-line chemo-naïve; PR+ = favorable predictor	CDK4/6i + ET active in ER-low advanced BC 1st-line; PR+ selects benefit	n=54; no RCT control	Partial/context-dependent
Sanford et al. (12)	Retrospective	Early BC	ER low/PR low HER2−	High BRCA1 germline mutation incidence in ER-low/PR-low/HER2− tumors	Germline BRCA testing mandatory; olaparib preferred over CDK4/6i in carriers	Referral population; not population-representative	Uncertain/heterogeneous
Malhlouf et al. (8)	Retrospective	Early BC	IHC 1–9%	Gene expression: ~75–80% non-luminal; luminal A/B only ~20–25%	PAM50 subtyping to identify minority with true luminal biology	Single institution; archival material	No benefit/TNBC-like
Allison et al. (7)	Guideline	Guideline	ER 1–10% → ‘ER Low Positive’	Formalized ER Low Positive category; limited ET benefit acknowledged	Explicit pathology comment required; 1% alone insufficient for ET decision	Consensus; uncertainty at 10% boundary	Uncertain/heterogeneous
Schmid et al. (29)	RCT	Neoadjuvant TNBC/ER-low	Molecular TNBC reclassification	Pembrolizumab + chemo: improved pCR and EFS in TNBC; ER-low basal-like may qualify	Evaluate immunotherapy eligibility when PAM50 = basal-like (PD-L1 CPS ≥10)	ER-low not pre-specified subgroup	Benefit (TNBC framework)

pCR, pathological complete response; sTIL, stromal TIL; NAC, neoadjuvant chemotherapy; MDT, multidisciplinary team; TMA, tissue microarray.

As shown in **Table 2**, three CDK4/6i have received regulatory approval for BC. Although they share the CDK4/6 inhibitory mechanism, they differ in selectivity, pharmacokinetics, dosing schedules, and off-target effects, factors that may be relevant to their relative efficacy in ER-low disease.

**Table 2:** CDKI4/6 inhibitors approved for breast cancer.

CDKI agent	Drug selectivity	Schedule	Trial name	Setting	Outcomes
Palbociclib	CDK4/6 selective	21 days on/7 days off (125 mg)	PALLAS, PENELOPE-B	Metastatic only	No benefit (class-wide negative)
Ribociclib	CDK4/6 selective	21 days on/7 days off (400 mg adjuvant)	NATALEE	Early BC (approved 2023)	Exploratory benefit maintained
Abemaciclib	CDK4/6 > CDK9, CLK2	Continuous twice daily (150 mg)	MONARCH E	High-risk early BC (approved 2021)	Numerically favorable HR ~0.65–0.78

ABE continuous dosing and broader kinase profile may confer distinct advantages in aggressive subtypes. Its more potent CDK4 inhibition relative to CDK6 may be mechanistically favorable in tumors where CDK4 activity predominates. The continuous schedule avoids potential cell cycle re-entry during drug holidays, particularly relevant in rapidly proliferating ER-low tumors. RIB consistent OS benefit in metastatic disease (MONALEESA-2, -3, -7 trials) has strengthened the rationale for early-stage investigation (30–33).

## 4 Clinical evidence: adjuvant CDK4/6 inhibitor trials

**Table 3** shows a comparative summary of major adjuvant CDK4/6i trials and ER-low relevant findings.

**Table 3:** Comparative summary of major adjuvant CDK4/6 inhibitor trials and ER-low relevant findings.

Trial	Schedule	Setting	Results	Conclusions
MONARCH E	Abemaciclib 2 yrs	High-risk HR+/HER2− (node+)	0.680–0.842 (OS) ✓ POSITIVE	Numerically favorable HR ~0.65–0.78; wide CI
NATALEE	Ribociclib 3 yrs	Stage IIA–III HR+/HER2− (broader)	0.716–0.748 ✓ POSITIVE	Exploratory benefit maintained across ER subgroups
PALLAS	Palbociclib 2 yrs	Stage II–III HR+/HER2−	0.96 ✗ NEGATIVE	No benefit across ER expression categories
PENELOPE-B	Palbociclib 1 yr	HR+/HER2−, residual disease post-NACT	0.93 ✗ NEGATIVE	No benefit; RB1 loss enriched in population

### 4.1 MONARCH E: abemaciclib in high-risk early HR+/HER2-/breast cancer

The MONARCH E (NCT03155997) phase III trial established ABE as a treatment option after initial therapy in HR+/HER2-/BC (5, 34, 35). Patients were eligible if they had at least four positive lymph nodes, or one to three positive nodes with either grade 3 tumors or tumors at least 5 cm (Cohort 1), or one to three positive nodes with lower-grade and smaller tumors but a Ki-67 of at least 20% (Cohort 2). Participants received standard ET with or without ABE 150 mg twice daily for two years. In 2023, the FDA removed the requirement for Ki-67 from the label. After a median follow-up of 6.3 years, ABE plus ET showed a significant improvement in OS (HR 0.842; 95% CI 0.722–0.981; p=0.027). Seven-year invasive disease-free survival (IDFS) was 77.4% with ABE versus 70.9% without (a 6.5% difference), and disease-recurrence-free survival (DRFS) was 80.0% versus 74.9% [50]. The ongoing benefit after treatment may suggest lasting immune effects or the elimination of small groups of cancer cells. In the MONARCH E trial, HR+ patients were included based on local pathology (ER ≥1% or PR ≥1%), including those with low ER (1–9%). This ER-low group accounted for about 3–5% of participants and had hazard ratios for IDFS with ABE (HR range 0.65–0.78), similar to the overall results, though the wide confidence intervals suggest the findings are not conclusive. The Ki-67 ≥20% rule for Cohort 2 may have included more ER-low patients, which could explain the consistent benefit observed, even if the biology differs.

### 4.2 NATALEE trial: ribociclib in intermediate- and high-risk early HR+/HER2- breast cancer

The NATALEE trial (NCT 03701334) tested RIB 400 mg daily (21 days on, 7 days off) for 36 months, plus nonsteroidal aromatase inhibitors (NSAI), compared with NSAI alone in a broad population of patients with stage IIA–III HR +/HER 2-/BC, including some node- negative (N 0) patients with additional risk factors (36–38). After a median follow-up of 55. 4 months for invasive disease- free survival (IDFS), RIB plus NSAI showed a lasting benefit (HR 0. 716; 95% CI 0. 618–0. 829), with the absolute IDFS improvement increasing from 2. 2.7% at 3 years to 4. 4.5% at 5 years. In the ER-low subgroup, the hazard ratio was 0.606 (95% CI 0.372–0.986). Updated overall survival (OS) data showed a trend favoring RIB (HR 0.800; 95% CI 0.637–1.003; p = 0.026) as more data become available [51]. The trial’s broad criteria included patients with lower ER levels. Exploratory analyzes by ER level suggest that the IDFS benefit with RIB was observed even in patients with 1–9% ER expression, with hazard ratios similar to those in the main results. While these findings are exploratory and not statistically powered, they represent some of the strongest evidence for CDK 4/6 inhibitor activity in ER- low disease. The three-year RIB treatment is longer than the two-year ABE regimen, which may provide more durable cell-cycle inhibition. This longer treatment could be important in ER-low tumors, where growth independent of CDK 4/6 may be more common.

### 4.3 PALLAS and PENELOPE-B trials: negative palbociclib evidence

The PALLAS trial (NCT 02513394) evaluated PAL for 2 years in stage II–III early HR+/HER2-/BC. It did not show an IDFS benefit (HR 0.96; 95% CI 0.81–1.14; p = 0.65) (39, 40). Exploratory analyzes in ER- low patients also found no differential benefit. The PENELOPE-B study (NCT 01864746) targeted HR+/HER2+ patients with residual disease after neoadjuvant chemotherapy (41, 42). This group is enriched for aggressive, treatment-resistant biology. It similarly yielded negative results (HR 0.93; 95% CI 0.74–1.17; P = 0.525) [8- PENELOPE]. The high proportion of RB1-deficient tumors in this group may have attenuated the effects of CDK4/6i.

The different results seen with PAL compared to ABE or RIB may be due to differences in how these drugs work. PAL is given in cycles and has a different kinase selectivity, with possibly weaker CDK4 activity and more off-target effects than ABE or RIB, which are given more continuously and have different kinase inhibition profiles. These differences can affect how well the drugs work and their side effects. Therefore, the negative results with PAL should not be applied to the whole drug class or to ER-low patients treated with ABE or RIB.

## 5 Biomarkers and patient selection in ER-low disease

### 5.1 RB1 status

Loss of RB1 function, through deletion, mutation, or epigenetic silencing, is both a mechanism of CDK4/6i resistance and a marker of aggressive tumor biology (43, 44). RB1 loss occurs in an estimated 20-30% of ER-low tumors, compared with <5% of ER-high luminal cancers, explaining why a meaningful subset of ER-low patients may not respond. Assessment of RB1 by IHC or NGS could identify primary resistance, though robust prospective validation in the adjuvant setting is currently lacking.

### 5.2 Ki-67 and proliferative index

High Ki-67 was an eligibility criterion in MONARCH E Cohort 2 and is associated with sensitivity to CDK4/6i (45). ER-low tumors tend to have higher baseline Ki-67, a feature that may paradoxically predict greater absolute benefit from CDK4/6i while reflecting a more aggressive phenotype (46). Ki-67 assessed at diagnosis or after a short-course neoadjuvant ET (as in the POETIC and ADAPT-Cycle strategies) may inform ER-low patient selection (47, 48).

### 5.3 Gene expression profiling and intrinsic subtypes

PAM50 intrinsic subtyping is particularly informative in ER-low disease: tumors classified as luminal A or luminal B may retain sensitivity to CDK4/6 inhibitors, whereas those classified as basal-like are unlikely to benefit from endocrine-directed strategies (49). *Post hoc* monarchE analyzes demonstrate consistent benefit from CDK4/6 inhibitors across luminal A and luminal B subtypes, with luminal B (predominant in ER-low disease) potentially deriving greater absolute benefit due to higher baseline risk. High Oncotype DX RS (≥26) is associated with TNBC-like biology in ER-low tumors; 67% of ER-low patients in a National Cancer Database analysis had RS ≥26 (15).

### 5.4 CCND1 amplification

CCND1 amplification at chromosome 11q13 occurs in 15–20% of BC, constitutively elevating cyclin D1 independent of ER signaling and potentially preserving CDK4/6i sensitivity in ER-low tumors (50, 51). Small studies in metastatic disease suggest that CCND1-amplified tumors derive greater benefit from CDK4/6i, though prospective adjuvant validation in ER-low disease is lacking (50, 51).

### 5.5 ctDNA and minimal residual disease

Circulating tumor DNA (ctDNA) as a minimal residual disease (MRD) marker has emerged as a powerful tool in early BC. Patients with detectable ctDNA postoperatively face a dramatically elevated risk of recurrence. ctDNA-guided therapy escalation, including addition of CDK4/6i, is being evaluated in early HR+/BC (52). This approach is conceptually attractive in ER-low disease, where the boundary between endocrine-responsive and endocrine-resistant biology is uncertain. The LEADER trial and ctDNA-integrated studies of ABE and RIB will provide prospective data (53, 54).

## 6 Treatment modalities in ER-low breast cancer

### 6.1 Endocrine therapy: evidence and open questions

Most guidelines report recommendation for ET for ER-low+ tumors (55). Still, meta-analyzes and large studies show that ET does not significantly improve survival, especially in the ER-low group (56, 57). One meta-analysis of about 10,000 patients found that ER-high patients had better survival than ER-low patients, but there was no survival difference between ER-low and ER-negative tumors (58). Another review of 16,000 patients found that ER-low+ patients treated with ET had outcomes similar to those not receiving ET (P = 0.684) and to ER-negative patients given ET (P = 0.145) (59).

Choong et al. recently found that skipping adjuvant ET was linked to shorter OS (HR 1.40; P < 0.001; adjusted HR 1.22; p = 0.05) in 10,396 ER-low+ patients (60). These results suggest that ET may offer survival benefits, possibly through pathways other than the estrogen receptor. For now, ET should not be routinely left out until more targeted studies are available. When ET is used, aromatase inhibitors are preferred for higher-risk ER+ disease, whereas tamoxifen should be avoided for RIB due to the risk of QTc prolongation. The best duration of ET in ER-low disease, such as 5 versus 10 years, has not been studied.

### 6.2 Neoadjuvant chemotherapy

ER-low+ BC patients have higher complete response rates to neoadjuvant chemotherapy (NAC) than ER-high patients (9, 61–64). Their outcomes are similar to those of patients with ER-negative tumors. A systematic review and meta-analysis confirmed that ER-low+ BC had higher pCR rates with NAC than ER+ tumors, and that these rates were similar to those in ER-negative tumors (62). Chen et al. found that ER-low+ patients had much higher pCR rates than those with ER expression above 10% (OR 0.249; p = 0.038) and had DFS and OS outcomes similar to those of ER-negative tumors (61). In a cohort of 358 patients, pCR was observed in 49.2% of TNBC and 51.3% of ER-low+ BC treated with NAC (63). These findings support the inclusion of ER-low+ BC patients in TNBC-focused NAC protocols. Suitable regimens include anthracycline/taxane-based treatments, with or without platinum agents, and immunotherapy when PD-L1 or TIL criteria are met.

### 6.3 Immunotherapy and emerging strategies

ER-low+ tumors often have higher levels of sTILs, CD8+ T cells, and PD-L1, supporting the use of immune checkpoint inhibitors (26, 65, 66). Luminal BC with high cell growth, low ER, and a high mitotic kinase score on Oncotype DX has a poor outlook, does not respond well to ET or CDK4/6 inhibitors, but is sensitive to chemotherapy (67, 68). These tumors also have higher rates of TP53 (34%) and PIK3CA (33%) mutations, a greater tumor burden, more TILs, and signs of PAL resistance (69, 70). The pCR rate to NAC is higher than in ER-rich tumors (22% vs 8%; p=0.06), but 4-year metastasis-free survival is lower (70% vs 94%; p=0.01).

ER-low+ BC is often excluded from TNBC immunotherapy antibody-drug conjugate trials, limiting the available evidence. The TROPION-Breast04 phase III trial is the first to include both ER-low+/HER2- breast cancer patients and TNBC (71). It is testing neoadjuvant datopotamab deruxtecan plus durvalumab. BRCA1 mutations are common in ER-low+/PR-low+/HER2- tumors. PARP inhibitor treatments, such as olaparib, should be systematically studied in both BRCA-mutant and possibly HRD-positive, non-BRCA tumors (12, 72, 73).

### 6.4 Safety and tolerability of CDK4/6 inhibitors

Medical oncologists need to consider the side effects of CDK4/6 inhibitors in patients with ER-low tumors (74). These patients are often younger, since ER-low is more common in premenopausal women. They may also receive chemotherapy or ovarian suppression concurrently. ABE causes more severe diarrhea (about 8%) due to its effects on CDK9 and the gut lining, so proactive management is important. PAL and RIB cause more neutropenia, which may require dose adjustments, but serious infections are rare. RIB can cause QTc prolongation and requires ECG monitoring, whereas ABE does not. In ER-low patients, balance the side effect risks of both chemotherapy and CDK4/6i. Also, keep in mind that treating as HR+ may miss TNBC-targeted therapies that could be more effective.

## 7 Eligibility thresholds, ER-low representation, and regulatory context

**Table 4** shows eligibility thresholds, ER-low representation, and regulatory context (75, 76). Both MONARCH E and NATALEE trials required locally confirmed HR+/HER2-/BC per institutional IHC, without specifying a minimum ER percentage. Because the 2010 ASCO/CAP threshold (≥1%) was widely adopted across the 38 countries (MONARCH E) and 20 countries (NATALEE) enrolling patients, ER-low patients (1–9%) were technically eligible for both trials. Neither trial reported quantitative subgroup analyzes by ER status.

**Table 4:** Patients’ eligibility thresholds, ER-low representation, and regulatory context.

Parameter	MONARCH E	NATALEE
HR+ definition	Local IHC, no ER% floor specified	Local IHC, no ER% floor specified
Nodal requirement	Node-positive only (≥1 pathological LN)	N0 with risk factors OR N1–3
Eligibility approach	Risk-feature based (nodes + grade/size/Ki-67)	AJCC anatomical stage IIA–III
Stage IIA N0	Not eligible	Eligible with high-risk features
Treatment duration	2 years abemaciclib	3 years ribociclib 400 mg/day
ET partner	NSAI or tamoxifen (~30% tamoxifen)	NSAI only (letrozole or anastrozole)
Real-world eligibility estimate	~14–22% of HR+/HER2− EBC patients	~31–43% of HR+/HER2− EBC patients
FDA approval	October 2021	September 2023

The FDA label for ABE does not restrict use to ER ≥10% or higher thresholds; ER-low patients meeting other eligibility criteria are technically eligible. Similarly, RIB prescribing information uses HR ≥1% as the minimum threshold. Major oncology guidelines (ASCO, NCCN, ESMO, St. Gallen 2023) have incorporated CDK4/6 inhibitors into adjuvant algorithms for high-risk HR+ EBC without specific exclusion of ER-low patients, while acknowledging the lack of prospective data in this subgroup (77).

## 8 Clinical decision-making framework

**Figure 1:**
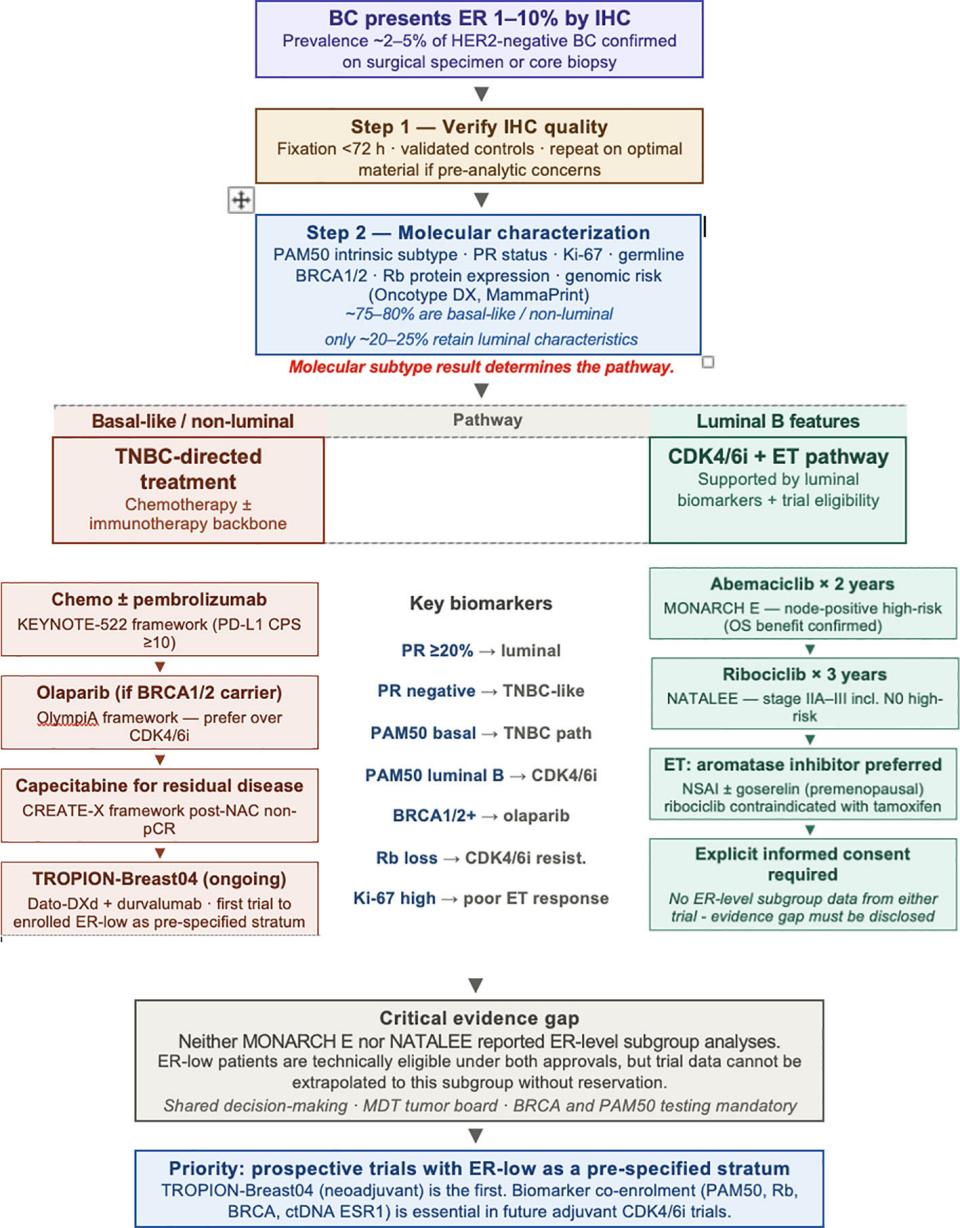
ER-low+ breast cancer: from diagnosis to treatment decision.

**Figure 1** provides a conceptual overview of the diagnostic and therapeutic pathway for ER-low+ patients, from tumor presentation through molecular characterization to treatment decision — encompassing both the TNBC-directed pathway for basal-like/non-luminal tumors and the CDK4/6i + ET pathway for the minority with true luminal biology, with key biomarkers guiding decisions at each step.

### 8.1 Stepwise clinical decision-making for ER-low patients meeting trial eligibility

**Table 5** depicts a stepwise framework proposal for ER-low patients meeting MONARCH E or NATALEE eligibility criteria.

**Table 5:** Structured clinical decision-making framework for ER-low patients meeting CDK4/6 inhibitor trial eligibility.

	Decision-Making needs	Conclusions
1	Verify IHC quality: confirm fixation <72 h, validated controls. Repeat if pre-analytical concerns.	Poor fixation is the most common cause of falsely low ER; technical artefact must be excluded first.
2	Assess PR status and Ki-67. PR ≥20% and luminal B Ki-67 profile support HR+ classification; PR-negative and high Ki-67 point toward TNBC-like biology.	PR positivity is the strongest indicator of residual luminal biology in ER-low tumors.
3	Perform molecular subtyping (PAM50/Prosigna or equivalent). Basal-like or HER2-enriched result redirects management toward TNBC-directed strategies.	~75–80% of ER-low tumors are non-luminal; subtyping resolves biological uncertainty.
4	Assess RB1 by IHC or NGS. RB1 loss predicts CDK4/6 inhibitor resistance; present in ~20–30% of ER-low tumors.	RB1 function is a prerequisite for CDK4/6 inhibitor activity; loss warrants redirecting to TNBC pathway.
5	Test for germline BRCA1/2. In BRCA carriers meeting OlympiA criteria, olaparib is preferred over CDK4/6 inhibitor.	High BRCA mutation prevalence in ER-low tumors; olaparib has superior evidence base in BRCA carriers.
6	Evaluate neoadjuvant chemotherapy response. RCB class II/III post-NAC suggests TNBC-like behavior; consider capecitabine (CREATE-X) and tumor board discussion.	pCR rates in ER-low BC are comparable to TNBC; non-pCR identifies high residual risk.
7	MDT tumor board: integrate all biomarker data. Luminal biology supported → offer CDK4/6i + ET with evidence gap counselling. Non-luminal → align with TNBC pathway.	No single biomarker suffices; integrated MDT assessment optimizes treatment selection.
8	Shared decision-making: inform patients that the ER-low subgroup was not prospectively studied in MONARCH E or NATALEE; extrapolation involves uncertainty.	Patients have the right to understand the limits of the evidence underpinning their treatment.

## 9 Future research directions

### 9.1 Dedicated ER-low adjuvant and neoadjuvant trials

Prospective trials with ER-low as a pre-specified stratum are warranted and overdue. Such trials should include a pre-specified ER-low analysis with adequate statistical power, centralized IHC pathology review, quantitative ER assessment (H-score or digital pathology quantification), and mandatory biomarker co-enrollment (PAM50, RB1 IHC, CCND1, germline BRCA, ctDNA). TROPION-Breast04 is the first such effort in the neoadjuvant setting. In the adjuvant setting, future CDK4/6 inhibitor trials should either specify a minimum ER threshold (≥10%) to ensure biological coherence or pre-specify ER-low as a separate analytical stratum (71).

### 9.2 CDK4/6 inhibition combined with TNBC-directed strategies

Given the biological overlap between ER-low and TNBC, including higher immune infiltration, tumor mutational burden, and BRCA-like features, there is a scientific rationale for evaluating CDK4/6i alongside TNBC-directed therapies, such as immune checkpoint blockade, PARP inhibitors, and antibody-drug conjugates (T-DXd, Dato-DXd) (26, 65). The immunomodulatory properties of CDK4/6i may synergize with PD-L1/PD-1 blockade in ER-low tumors (43).

### 9.3 ctDNA-guided therapy and resistance mechanisms

Liquid biopsy (ctDNA) for ESR1 mutations and MRD monitoring should be systematically evaluated in ER-low patients progressing on ET-based regimens. ESR1 mutations (detected in 40–50% of progressive ER+ BC) are targetable with elacestrant and imlunestrand (78); NCCN and ESMO guidelines already recommend ESR1 testing in progressive metastatic settings (79, 80). CCNE1 amplification-driven CDK2 bypass of RB1 arrest, activation of the PI3K/Akt/mTOR pathway, and PKMYT1 overexpression represent additional resistance mechanisms warranting dedicated investigation in ER-low disease (81–83).

### 9.4 Artificial intelligence and digital pathology

Quantitative digital pathology approaches and artificial intelligence-driven IHC analyzers improve the precision of ER characterization. Jung et al. demonstrated that AI-augmented interpretation of ER, PR, and HER2 significantly improved interobserver agreement in a reader study of 201 cases (82). Machine learning algorithms that integrate spatial ER expression patterns, tumor heterogeneity, and immune infiltration may identify predictive biomarkers of CDK4/6 inhibitor response in ER-low disease. Integrating these approaches with transcriptomic and genomic data in large retrospective trial databases is a priority research direction (84).

## 10 Conclusions

CDK4/6i have demonstrated transformative benefit in adjuvant early HR+BC, as confirmed by an OS benefit at 6 years in the MONARCH E trial and a persistent IDFS benefit at 5 years in the NATALEE trial. ER-low BC is a biologically heterogeneous, clinically high-risk subset in which CDK4/6i benefit is biologically plausible but not yet definitively established by prospective data. The available evidence is of low to moderate quality and largely retrospective or exploratory. Exploratory data from MONARCH E (HR ~0.65–0.78) and NATALEE (consistent benefit across ER expression categories) suggest a numerically consistent CDK4/6i benefit in ER-low subgroups; however, the confidence intervals are wide, and formal statistical significance is not demonstrated. The negative PAL trials do not specifically inform the ER-low question for the class, reflecting agent-specific pharmacology.

From a clinical practice standpoint, ER-low patients meeting high-risk MONARCH E or NATALEE criteria may receive adjuvant CDK4/6i when supported by luminal biomarkers, particularly PR positivity, intact RB1, Luminal B PAM50 subtype, and elevated Ki-67, following MDT discussion and explicit informed consent about the evidence gap. When molecular profiling confirms basal-like biology, TNBC-directed strategies, such as chemotherapy with or without immunotherapy, olaparib in BRCA carriers, and capecitabine for residual disease, should be prioritized. The ER-low population deserves dedicated clinical investigations. Prospective trial designs that enrich for this subgroup and integrate robust molecular profiling and ctDNA-based MRD monitoring are essential to resolving the CDK4/6i question and advancing precision oncology at the uncertain boundary between luminal and basal-like BC.
